# Correction to: Interpreting UniFrac with absolute abundance: a conceptual and practical guide

**DOI:** 10.1093/ismeco/ycag078

**Published:** 2026-05-19

This is a correction to: Augustus Pendleton, Marian L Schmidt, Interpreting UniFrac with absolute abundance: a conceptual and practical guide, *ISME Communications*, Volume 6, Issue 1, January 2026, ycaf250, https://doi.org/10.1093/ismeco/ycaf250

The supplementary data has been corrected to include a single file. The previously published pdfs: **Supporting_Information_ycaf25002_Pendleton_ISME_PostReview_SupportingInformation_Clean_ycaf250** and **Pendleton_ISME_PostReview_SupportingInformation_ycaf250** have been removed.

