# Supplementary material for: Interpreting UniFrac with absolute abundance: a conceptual and practical guide
Source: ISME Commun. 2026 Jan 6;6(1):ycaf250. doi: 10.1093/ismeco/ycaf250 (PMC12900076; doi:10.1093/ismeco/ycaf250)
Supplement: 03_Pendleton_Supplement_FINAL_ycaf250 [file 03_pendleton_supplement_final_ycaf250.pdf]

Supporting Information

Interpreting UniFrac with Absolute Abundance: A Conceptual and Practical Guide

Augustus Pendleton[1]* & Marian L. Schmidt[1]*

[1]Department of Microbiology, Cornell University, 123 Wing Dr, Ithaca, NY 14850, USA

Corresponding Authors: Augustus Pendleton: arp277@cornell.edu; Marian L. Schmidt: marschmi@cornell.edu; MarianL.Schmidt@gmail.com

Supporting Methods

ASV Generation and Phylogenetic Tree Construction

Sequencing data and identifying metadata were downloaded from the Sequence Read Archive (SRA), from BioProject IDs PRJNA815056, PRJNA575097, PRJNA1212049, and PRJNA302180 [1–4]. Full details and code of the Pendleton et al. 2025 data analysis are included within that paper and associated Github repository and not shown here. Each dataset varied substantially in terms of which 16S region it targeted, sequencing strategy, and read quality, so ASV generation varied between them in terms of primer removal, filtering, and trimming (see code for full description of these steps). Post trimming, all ASVs were generated using the same methods within the standard DADA2 workflow [5]. Chimaeras were removed, and ASVs were size selected (252/253 bp for V4 datasets, >400bp for V3-V4 datasets). Taxonomy was assigned via the Silva v138.2 database, and used to remove mitochondrial and chloroplast sequences [6]. When sequencing positives or negatives were present, they were removed.

Phylogenetic trees were built using alignment via MAFFT followed by FastTree under a generalized time-reversible model [7, 8]. Trees were visualized via ggtree in R, and anomalously long branches were removed using ape [9]. Trees, metadata, taxonomy, and ASV abundances (OTU tables) were organized and analyzed using phyloseq [10].

Rarefaction and β-diversity

To generate rarefied ASV tables of equal sequencing depth, ASV abundance matrices were subsampled using a multivariate hypergeometric distribution via the rmvhyper function in the extraDistr package (see generate_rarefied_abs_tables.R) [11]. Each ASV was then converted to relative abundances, and then to absolute abundances by multiplying the relative abundance by each samples cell count or 16S copy number. Bray-Curtis dissimilarities were calculated via the vegdist function in vegan [12]. Unless otherwise noted, all Unifrac distances were calculated via the GUnifrac package [13]. Final distance matrices were the average of all rarefied distance matrices. All samples within each dataset were used for contour plots in Figure 2.

For the analysis of rarefaction itself (Fig. S4), datasets were rarefied to multiple sequencing depths (250, 500, 1000, 5000, 10,000 reads per sample), as well as the minimum sequencing depth observed in each dataset (ranging from 1,086 reads per sample in the cooling reactor dataset to 48,601 reads per sample in the soil dataset). In addition, a non-rarefied control from each dataset was generated as a control. Absolute abundance normalization and GU^A calculation were then performed as described above.

PERMANOVAs, Ordinations, and Mantel Tests

PERMANOVAs were conducted via the adonis2 function in vegan (Fig. 3 and Fig. S2). To limit confounding variables, not all samples were used in these analyses. From the cooling water dataset, just samples from Reactor cycle 1 were used. For the mouse gut, just stool samples were used. For the soil dataset, just mature samples were used. All PERMANOVAs were run with 1,000 iterations. These same, simplified datasets were used for Principal Coordinates Analysis in Fig S3. When estimating the correlation between distance matrices, we used the mantel function from the vegan package with 999 permutations.

Timing Analysis

To estimate computational time, we subsampled the soil dataset to a set number of ASVs, samples, and α numbers. When testing ASV number, we used 10 samples and one α value, when testing sample or α values, ASVs were held constant at 2,000. Each case was replicated 20 times, and computation time was calculated via the microbenchmark function from the microbenchmark package, with two replications each time [14].

Error Analysis

To estimate the impact of random error on quantification methods, we used the mouse gut dataset, focusing only on the stool samples. These samples ranged in 16S copy number from 10^{11}-10^{12} copies/gram. We tested a range of potential error, from 1% up to 50%. For each error percentage, the amount of error was selected from a normal distribution with a mean of that error percent and a standard deviation 1/10th of that error percentage. This error was then randomly assigned a direction (by multiplying by a binomial distribution of -1 and 1), and multiplied by the copy number to create a deviation from the true value, which was added to the original value. For example, in the case of 50% error, we first drew a random selection of error values from a distribution with mean 0.5 and standard deviation of 0.05. These errors were then randomly assigned to be negative or positive, and multiplied by the original cell counts, plus the cell count itself. We repeated this fifty times. Across these fifty iterations, we first rarefied the ASV abundances to relative abundance and then normalized to absolute abundance using these error-added values. We then compared the absolute difference in GU^A or BC^A from these error-added datasets compared to the original data to produce Fig. 4C-D.

Copy Number Normalization

We used PICRUSt2 (v2.6, with the updated PICRUSt2-SC database) to normalize ASV abundances for each dataset, using the default settings (NSTI = 2) in the picrust2_pipeline.py function [15]. These normalized ASV tables (in /EC_metagenome_out/seqtab_norm.tsv.gz) were then used to calculate GU^A using our standard pipeline, including rarefaction to the minimum sequencing depth (across 10 iterations), normalization by absolute abundance, and averaging of GU^A across iterations. The predicted 16S rRNA gene copy number of each ASV (for Fig. S6) was accessed from combined_marker_predicted_and_nsti.tsv.gz.

Other Coding Packages

Other packages used for general coding and visualization include tidyverse, purr, patchwork, NatParksPalette, broom, corrr, ggpubr, biomformat, and renv. All packages and version numbers are listed in Table S1.

Supplemental Figures

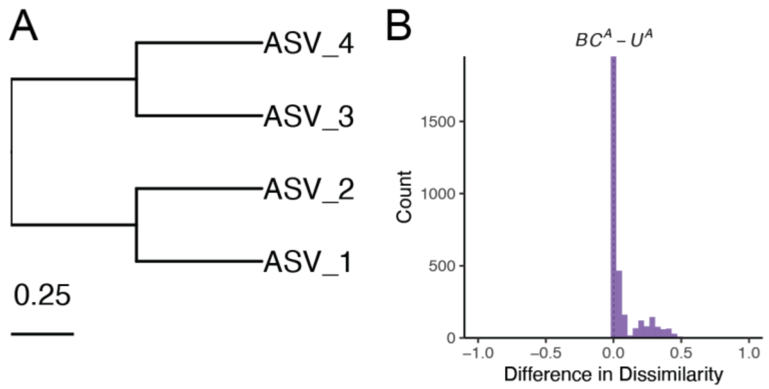

Figure S1. U^A is always less than BC^A when branch lengths are fully symmetrical. (A) Symmetrical tree used for simulations as opposed to non-symmetrical tree in Fig. 1A. (B) Distribution of differences between BC^A and U^A. As the differences are never negative, U^A is always less than or equal to BC^A.

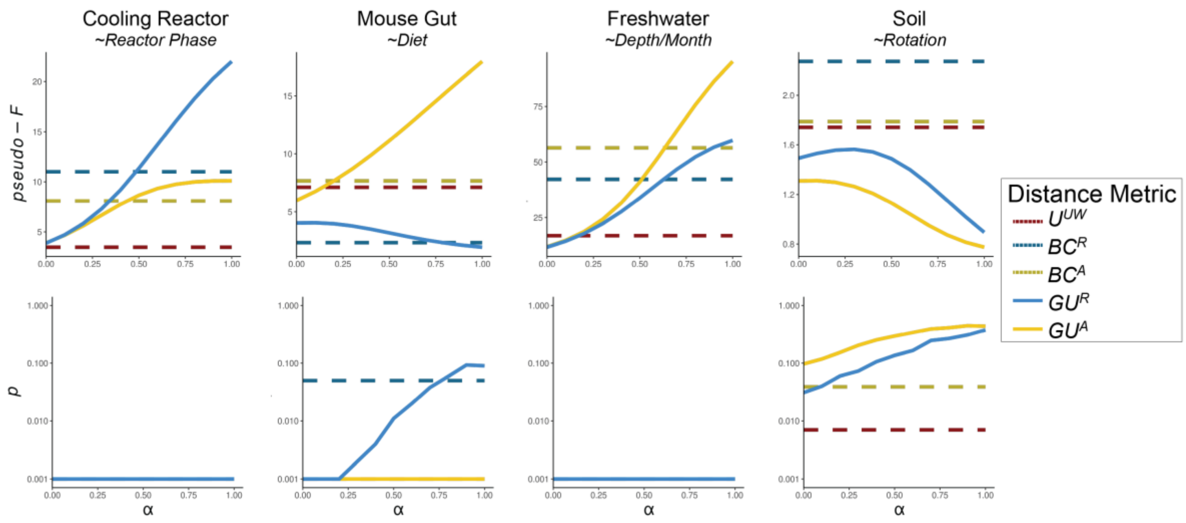

Figure S2. Additional PERMANOVA results when using GU^A across a range of α values. PERMANOVAs were run testing the significance of two-three category groups from each dataset (provided in italics beneath data names). Results indicate *pseudo-F* statistics and *p*-values after 1,000 iterations. In the cooling reactor, only samples from Reactor cycle 1 were used; in the mouse gut, only stool samples were used, and in the soil, only mature samples were used. Note the y-axes for *pseudo-F* plots are variable between datasets, and y-axes for the *p*-value plots are log-scaled.

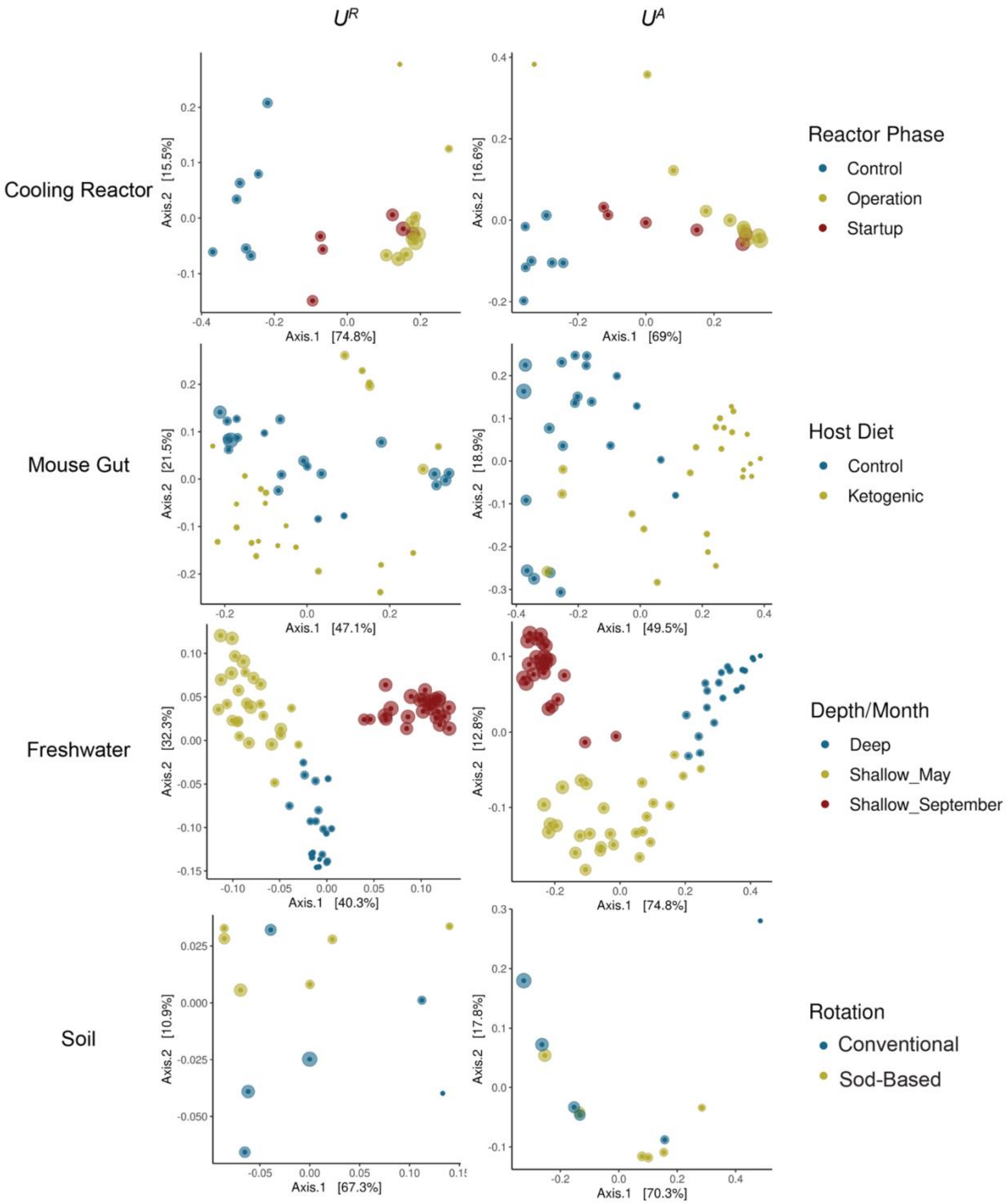

Figure S3. Principal Coordinate Analysis ordinations of each dataset using U^R and U^A. Points are colored using the same categorical variable tested in the PERMANOVAs of Fig. 2 and Fig. S2 (for additional details on experimental design, see [1, 3, 4, 16]). Both U^R and U^A were calculated at an $\alpha = 1$.

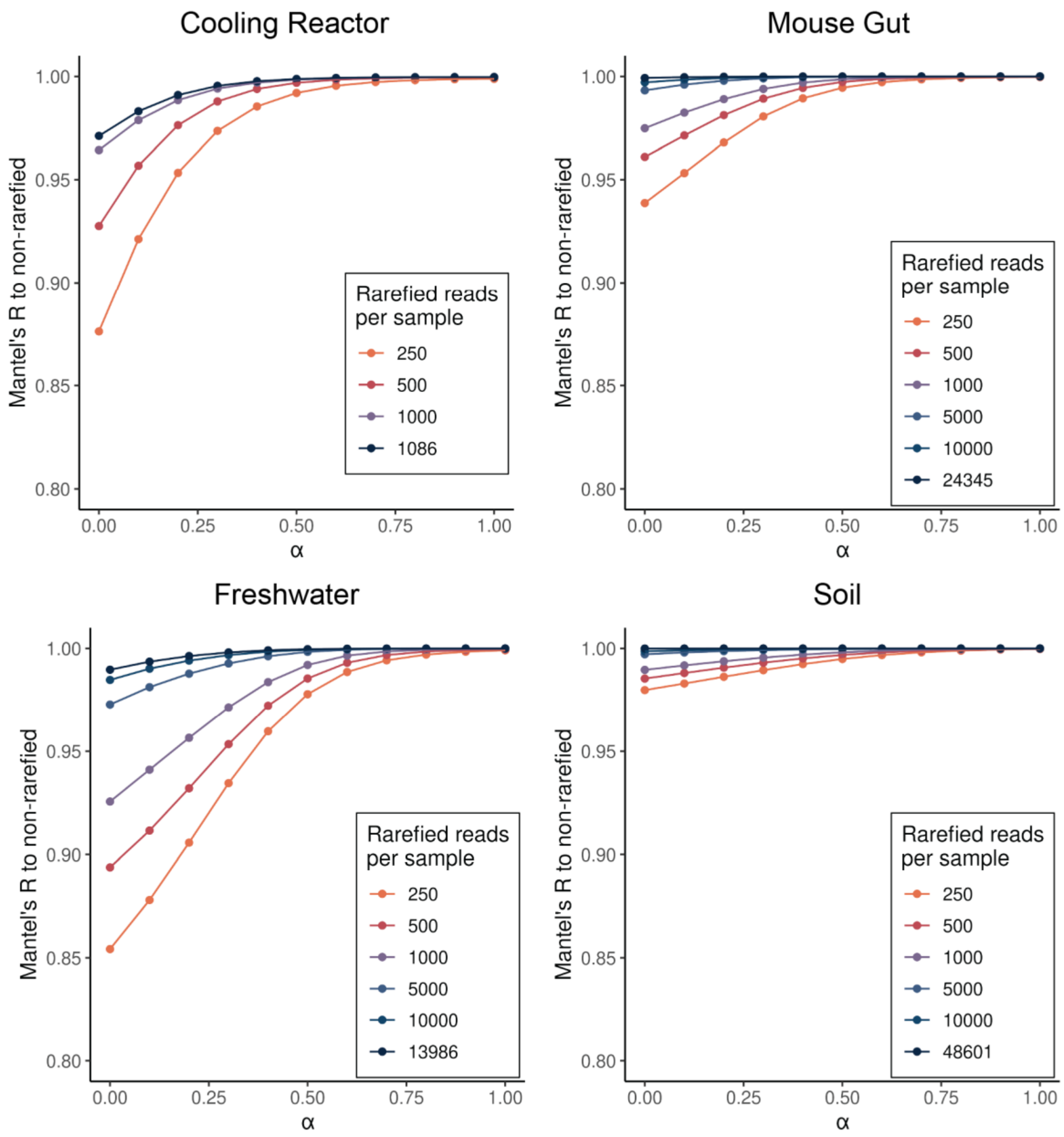

Figure S4. Effect of rarefaction on GU^A. GU^A was calculated across α values from 0 up to 1 at multiple rarefaction depths. Depths ranged from 250 reads/sample, up to minimum sequencing depth for each dataset (1,086 reads/sample in the cooling reactor, up to 48,601 reads/sample in the soil dataset). Mantel tests were then used to calculate the correlation between the rarefied GU^A distance matrices compared to GU^A calculated on the non-rarefied control. Each rarefaction depth was calculated across 10 iterations.

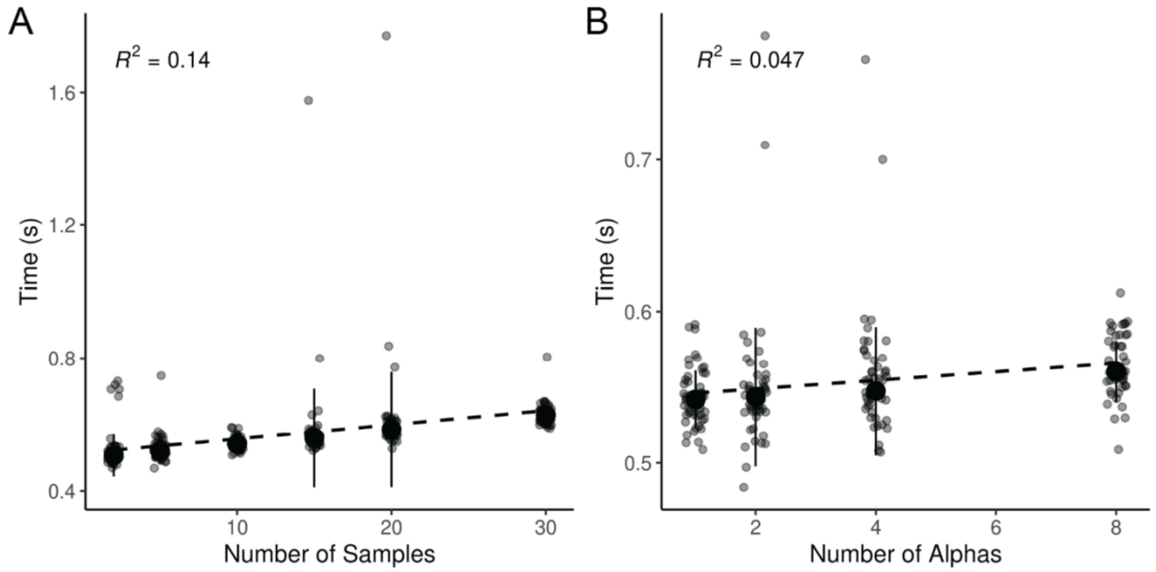

Figure S5. Additional parameters which weakly influence computation time for GU^A. A) GU^A was calculated 50 times across six sample sizes (2, 5, 15, 20, 30) with a constant of 2,000 ASVs and one calculated α (though unweighted Unifrac is also calculated by default). B) GU^A was calculated 50 times across four alpha parameter sizes (1, 2, 4, 8; note unweighted Unifrac is also calculated by defualt) with a constant of 2,000 ASVs and 10 samples. In both panels, R^2 is derived from a linear model between the x and y axes.

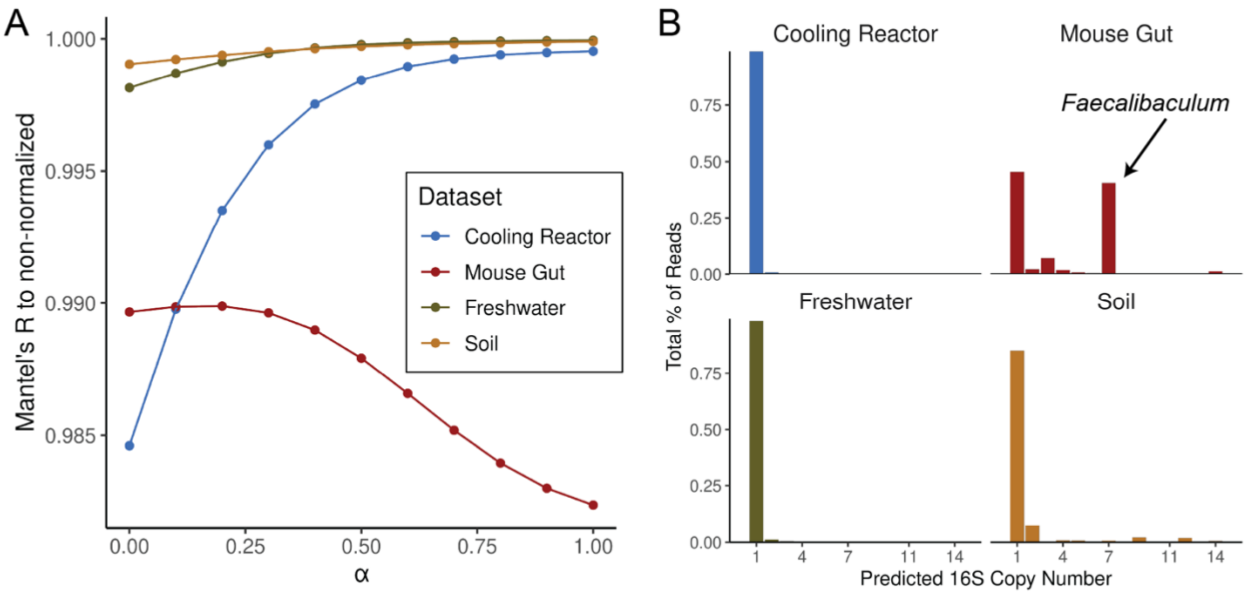

Figure S6. 16S rRNA gene copy number-normalization had a negligible effect on GU^A. A) PICRUSt2 (v2.6) was used to normalize sequencing reads within each sample by the predicted 16S rRNA gene copy number [15]. GU^A was then calculated across α values from 0 to 1. Mantel tests were used to assess correlations between the GU^A distance matrices calculated from copy number-normalized dataset and those calculated from non-normalized controls. For both analyses, rarefaction was carried out to the minimum sequencing depth for each dataset across 10 iterations. The y-axis is truncated to highlight the differences among correlations, all of which exceed a Mantel's R of 0.98. B) Percentage of total reads assigned to ASVs at each predicted 16S copy number. Predicted copy numbers extended up to 27 copies per genome, however, categories with negligible representation are not shown. In the mouse gut dataset, ASVs (and reads) with seven predicted 16S copies per genome primarily belonged to the genus *Faecalibaculum*, with one ASV assigned to *Escherichia-Shigella* and one to the class Clostridia.

Package/Software	Version	Citation
R	4.3.3	[17]
RStudio	2024.12.1+563	[18]
tidyverse	2.0.0	[19]
phyloseq	1.52.0	[10]
vegan	2.7-1	[12]
GUniFrac*	1.8.1	[13]
ggtree	3.16.0	[20]
patchwork	1.3.1	[21]
NatParksPalettes	0.2.0	[22]
ape	5.8-1	[9]
broom	1.0.8	[23]
corrr	0.4.4	[24]
renv	1.0.5	[25]
microbenchmark	1.5.0	[14]
ggpubr	0.6.1	[26]
dada2	1.36.0	[5]
MAFFT	7.520	[7]
FastTree	2.1.11	[8]
cutadapt	5.1	[27]
extraDistr	1.10.0	[11]
PICRUSt2	2.6.0	[15, 28]
biomformat	1.30.0	[29]

Table S1. Software and packages used in analysis. Note that GUniFrac was modified slightly to make incorporating absolute abundances more apparent; this version can be installed via Github at https://github.com/MarschmiLab/GUniFrac.

Supporting References

1. Zhang K et al. Absolute microbiome profiling highlights the links among microbial stability, soil health, and crop productivity under long-term sod-based rotation. *Biol Fertil Soils* 2022;**58**:883–901. https://doi.org/10.1007/s00374-022-01675-4
2. Props R et al. Measuring the biodiversity of microbial communities by flow cytometry. *Methods in Ecology and Evolution* 2016;**7**:1376–1385. https://doi.org/10.1111/2041-210X.12607
3. Pendleton A, Wells M, Schmidt ML. Upwelling periodically disturbs the ecological assembly of microbial communities in the Laurentian Great Lakes. 2025. bioRxiv, 2025. , 2025.01.17.633667
4. Barlow JT, Bogatyrev SR, Ismagilov RF. A quantitative sequencing framework for absolute abundance measurements of mucosal and lumenal microbial communities. *Nat Commun* 2020;**11**:2590. https://doi.org/10.1038/s41467-020-16224-6
5. Callahan BJ et al. DADA2: High-resolution sample inference from Illumina amplicon data. *Nat Methods* 2016;**13**:581–583. https://doi.org/10.1038/nmeth.3869
6. Quast C et al. The SILVA ribosomal RNA gene database project: improved data processing and web-based tools. *Nucleic Acids Research* 2013;**41**:D590–D596. https://doi.org/10.1093/nar/gks1219
7. Katoh K, Standley DM. MAFFT Multiple Sequence Alignment Software Version 7: Improvements in Performance and Usability. *Molecular Biology and Evolution* 2013;**30**:772–780. https://doi.org/10.1093/molbev/mst010
8. Price MN, Dehal PS, Arkin AP. FastTree 2 – Approximately Maximum-Likelihood Trees for Large Alignments. *PLOS ONE* 2010;**5**:e9490. https://doi.org/10.1371/journal.pone.0009490

9. Paradis E et al. ape: Analyses of phylogenetics and evolution. 2023.
10. McMurdie PJ, Holmes S. phyloseq: An R package for reproducible interactive analysis and graphics of microbiome census data. *PLoS ONE* 2013;**8**:e61217.
11. Wolodzko T. extraDistr: Additional univariate and multivariate distributions. 2023.
12. Oksanen J et al. vegan: Community ecology package. 2022.
13. Chen J et al. Associating microbiome composition with environmental covariates using generalized UniFrac distances. *Bioinformatics* 2012;**28**:2106–2113. https://doi.org/10.1093/bioinformatics/bts342
14. Mersmann O. microbenchmark: Accurate timing functions. 2024.
15. Wright RJ, Langille MGI. PICRUSt2-SC: an update to the reference database used for functional prediction within PICRUSt2. *Bioinformatics* 2025;**41**:btaf269. https://doi.org/10.1093/bioinformatics/btaf269
16. Props R et al. Absolute quantification of microbial taxon abundances. *ISME J* 2017;**11**:584–587. https://doi.org/10.1038/ismej.2016.117
17. R Core Team. R: A language and environment for statistical computing. Vienna, Austria: R Foundation for Statistical Computing, 2022.
18. RStudio Team. RStudio: Integrated Development Environment for R. Boston, MA: RStudio, PBC., 2020.
19. Wickham H. tidyverse: Easily install and load the tidyverse. 2023.
20. Xu S et al. Ggtree: A serialized data object for visualization of a phylogenetic tree and annotation data. *iMeta* 2022;**1**:e56. https://doi.org/10.1002/imt2.56
21. Pedersen TL. patchwork: The composer of plots. 2024.
22. Blake K. NatParksPalettes: Color palettes inspired by national parks. 2022.

23. Robinson D, Hayes A, Couch S. broom: Convert statistical objects into tidy tibbles. 2023.

24. Kuhn M, Jackson S, Cimentada J. corrr: Correlations in R. 2022.

25. Ushey K, Wickham H. renv: Project environments. 2024.

26. Kassambara A. ggpubr: ggplot2 based publication ready plots. 2023.

27. Martin M. Cutadapt removes adapter sequences from high-throughput sequencing reads. *EMBnet.journal* 2011;**17**:10–12. https://doi.org/10.14806/ej.17.1.200

28. Douglas GM et al. PICRUSt2 for prediction of metagenome functions. *Nat Biotechnol* 2020;**38**:685–688. https://doi.org/10.1038/s41587-020-0548-6

29. McMurdie PJ, Paulson JN. biomformat: An interface package for the BIOM file format. 2023.
